# Memory-efficient neurons and synapses for spike-timing-dependent-plasticity in large-scale spiking networks

**DOI:** 10.3389/fnins.2024.1450640

**Published:** 2024-09-06

**Authors:** Pablo Urbizagastegui, André van Schaik, Runchun Wang

**Affiliations:** International Centre for Neuromorphic Systems, The MARCS Institute for Brain, Behavior, and Development, Western Sydney University, Kingswood, NSW, Australia

**Keywords:** synaptic plasticity, large scale, neuromorphic computing, digital simulation, memory architecture

## Abstract

This paper addresses the challenges posed by frequent memory access during simulations of large-scale spiking neural networks involving synaptic plasticity. We focus on the memory accesses performed during a common synaptic plasticity rule since this can be a significant factor limiting the efficiency of the simulations. We propose neuron models that are represented by only three state variables, which are engineered to enforce the appropriate neuronal dynamics. Additionally, memory retrieval is executed solely by fetching postsynaptic variables, promoting a contiguous memory storage and leveraging the capabilities of burst mode operations to reduce the overhead associated with each access. Different plasticity rules could be implemented despite the adopted simplifications, each leading to a distinct synaptic weight distribution (i.e., unimodal and bimodal). Moreover, our method requires fewer average memory accesses compared to a naive approach. We argue that the strategy described can speed up memory transactions and reduce latencies while maintaining a small memory footprint.

## 1 Introduction

Evolution endowed neural systems with a level of complexity that is infeasible to emulate *in silico* without trade-offs. For instance, human brains contain 86 billion neurons, with a substantial metabolic cost of about 20% of the total body energy budget (Herculano-Houzel, 2012). In addition to that, many synaptic connections between neurons, which are a crucial part of neuroscience research, are not static; they are rather plastic entities that change according to activity, thereby altering the dynamics of the associated networks (Feldman, 2012). Aside from the sheer scale and dynamic nature of neural architecture, neurons operate continuously, processing and transmitting information in real time. Considering these complexities, alongside myriad other sources of intricacy within neural systems, it is unsurprising that simulating even a small, simplified subset of these networks on modern computers can become impractical, primarily due to the substantial memory requirements involved.

One of the central complexities we addressed in this work revolves around synaptic plasticity. Within this context, Spike-Timing-Dependent-Plasticity (STDP) holds a prominent position. STDP represents a simple yet powerful plasticity rule that reproduces numerous experimental findings in neurobiology (Bi and Poo, 1998; Markram et al., 2011). Nevertheless, its computational demands can present challenges when translated into the digital realm.

To illustrate this, consider a network comprising N neurons. In this scenario, an *N*×*N* crossbar structure is often employed to store connectivity variables, such as synaptic weights. When a neuron emits a spike, both outgoing and incoming connections may necessitate updates, contingent upon the spiking activities of post- and presynaptic neurons, respectively. Handling plasticity operations related to outgoing connections is straightforward since conventional memory arrays support parallel row-wise access (Seo et al., 2011; Knight and Nowotny, 2018). In contrast, the column-wise access (or “reverse” access, as opposed to “forward” access) linked with incoming synapses is inefficient, frequently relying on additional operations to pinpoint the connected presynaptic neurons (Alevi et al., 2022) or allocating separate data structures dedicated to these connections (Knight and Nowotny, 2018). These processes, however, contribute to increased computation time and expanded memory footprint (Pedroni et al., 2019).

Alternative crossbar architectures can be used to address these inefficiencies in column-wise access, thereby promoting scalability and on-chip learning (Seo et al., 2011; Frenkel et al., 2018). Nonetheless, using a synapse crossbar has a drawback: nonexistent connections within the network still occupy physical space. This compromises silicon area and is particularly problematic for sparse, recurrent networks (Pedroni et al., 2019). Instead of implementing synapse crossbars or introducing dependencies on additional state variables, alternative approaches focus on delaying weight updates. For example, in neuromorphic boards such as SpiNNaker, presynaptic spikes trigger acausal weight updates as usual, but causal updates due to postsynaptic spikes occur only when another presynaptic spike is delivered at the corresponding synapse (Diehl and Cook, 2014, but see Bogdan et al. (2020), for a more current implementation). With Loihi, Davies et al. (2018) defined a learning epoch time, after which plasticity takes place. Pedroni et al. (2019) pursued a similar approach and demonstrated that pointer-based data structures, such as compressed sparse row, serve as efficient alternatives for memory storage. More importantly, as previously shown, the inefficiencies associated with reverse access required by postsynaptic spikes can be circumvented by slightly delaying weight updates. In other words, both causal and acausal updates can be executed with forward access driven by presynaptic custom events, provided that spike time information is adequately stored. This strategy facilitates contiguous memory allocation, which leads to improved memory access and faster simulation of Spiking Neural Networks (SNNs; Bautembach et al., 2021). In essence, more efficient methods of accessing memory in digital systems have enabled a wealth of scalable and fast simulation platforms (Thakur et al., 2018; Frenkel et al., 2023).

Research in computational neuroscience deals with highly complex systems, so simulation strategies are not limited to improving memory access efficiency. Simplifications are often adopted to create tractable models that can be integrated into large network models (Teeter et al., 2018; Chen et al., 2022; Pagkalos et al., 2023). Importantly, simpler models can be exploited to optimize a design. For instance, a shared update logic (e.g., exponential decay) allows a time-multiplexing scheme, which leads to better resource utilization and scalability (Wang and van Schaik, 2018; Modaresi et al., 2023).

In this work, we delve into an innovative alternative to STDP for digital hardware that enhances efficiency and scalability and reduces memory footprint. We achieved this by enforcing contiguous memory allocation and a reduced model complexity. Specifically, we have devised a custom event mechanism that facilitates weight updates (causal and acausal) with forward access only. Since we were interested in the sparsity of more realistic neuronal connectivity, we tackled a pointer-based structure for weight storage. Furthermore, our investigation demonstrates the feasibility of reducing the number of state variables used per neuron for computing plastic changes, thus further increasing scalability. Finally, we considered the implications of this reduction on the network statistics.

## 2 Materials and methods

### 2.1 State variables

The simulations described here were carried out using Brian2 (Stimberg et al., 2019) with its graphics processing unit backend (Alevi et al., 2022). The equations governing the membrane potential (*V*_*m*_) and postsynaptic potential (PSP) are


(1) $$\begin{array}{l} V_{m}\left[t+1\right]=\alpha_{m}V_{m}\left[t\right]+\alpha_{m}dt\frac{\text{PSP}\left[t\right]}{\tau_{m}} \end{array}$$

and


(2) $$\begin{array}{l} \text{PSP}\left[t+1\right]=\alpha_{syn}\text{PSP}\left[t\right], \end{array}$$

respectively, where τ_*m*_ = 20 ms and τ_*syn*_ = 5 ms. We adopted α_*m*_ = τ_*m*_/(τ_*m*_+*dt*), α_*syn*_ = τ_*syn*_/(τ_*syn*_+*dt*), and *dt* = 1 ms. Equations 1, 2 represent simple dynamics of LIF neuron models and current-based synapses. A spike is generated if the *V*_*m*_>*V*_*thr*_ = 20 mV, in which case *V*_*m*_ is set to *V*_*reset*_ = 0 mV and the cell becomes refractory for 2 ms. A crucial distinction herein is that the “ownership” of state variables was engineered to minimize memory footprint. Specifically, instead of allocating one PSP for each synaptic connection, our model assigns one for each neuron.

A presynaptic spike from an excitatory (inhibitory) neuron *i* connected to a neuron *j* increments (decrements) the PSP value according to the synaptic weight *w*_*ji*_. Regarding synaptic plasticity, changes in the strength of excitatory-excitatory connections *w*_*ji*_ are regulated by the interaction between spikes and their timing information. This information is stored in traces. Similarly to other state variables, each neuron holds one trace *x*(*t*), which is increased by Δ*x* whenever this neuron emits an action potential. The evolution of *x* over time can be defined as


(3) $$\begin{array}{l} x\left[t+1\right]=\alpha_{x}x\left[t\right], \end{array}$$

where α_*x*_ = τ_*x*_/(τ_*x*_+*dt*).

### 2.2 Plasticity implementation

In conventional STDP, *w*_*ji*_ can be updated as


$$w_{ji}=\left\{ \begin{array}{l} w_{ji}-\eta x_{j}\;(4)\\ w_{ji}+\eta x_{i},\;(5) \end{array}\right.$$

with a learning rate η and state variable *x* as defined in Equation 3. Equation 4 is computed upon the occurrence of a presynaptic spike, while Equation 5 is evaluated whenever there is a postsynaptic spike. In other words, spikes must be detected and stored in memory. The state variables associated with those spiking neurons must then be fetched from memory so that acausal or causal updates can occur.

During our study, we signaled a spike by momentarily setting the most significant bit of a neuronal *x* trace to 1. This was accomplished in Brian2 by simply setting the trace to a negative value, but the positive sign had to be restored by the end of the simulation time step. Since we wanted to avoid the overhead of reverse memory access triggered by a postsynaptic spike, we explored a methodology that allows for weight updates exclusively through forward access. Essentially, whenever a presynaptic neuron was active (see definition below), all the postsynaptic traces were retrieved from memory. A negative presynaptic trace paired with a positive postsynaptic trace triggered acausal updates, whereas the opposite caused a causal update. No updates were performed when both neurons fired simultaneously.

In our simulations, we have defined a neuron as active when its *x* variable exceeds a certain threshold *x*_*thr*_. When a neuron spikes, the outgoing weights are tentatively updated, that is, weights change only under the right conditions. This process is repeated in subsequent time steps as long as the presynaptic trace remains above the designated threshold. If a postsynaptic spike eventually occurs, a causal update can take place. Notably, there is no necessity for reverse access to locate the presynaptic neurons; all active neurons are already triggering the necessary updates based on the temporal information carried by the traces.

Our scheme can be summarized as follows: As long as a neuron is active, outgoing weights are updated according to


(6) $$\begin{array}{l} w_{ji}=\left\{ \begin{array}{cc} w_{ji}-\eta x_{j} & \text{if }x_{i}<0\wedge x_{j}>0\\ w_{ji}+\eta x_{i}, & \text{if }x_{i}>0\wedge x_{j}<0 \end{array}\right. \end{array}$$

where ∧ is a simple AND logic operator. Note that we can cast the above equations as a conventional STDP rule if the first condition is “neuron *i* spiked” and the second is “neuron *j* spiked.”

**Figure 1:**
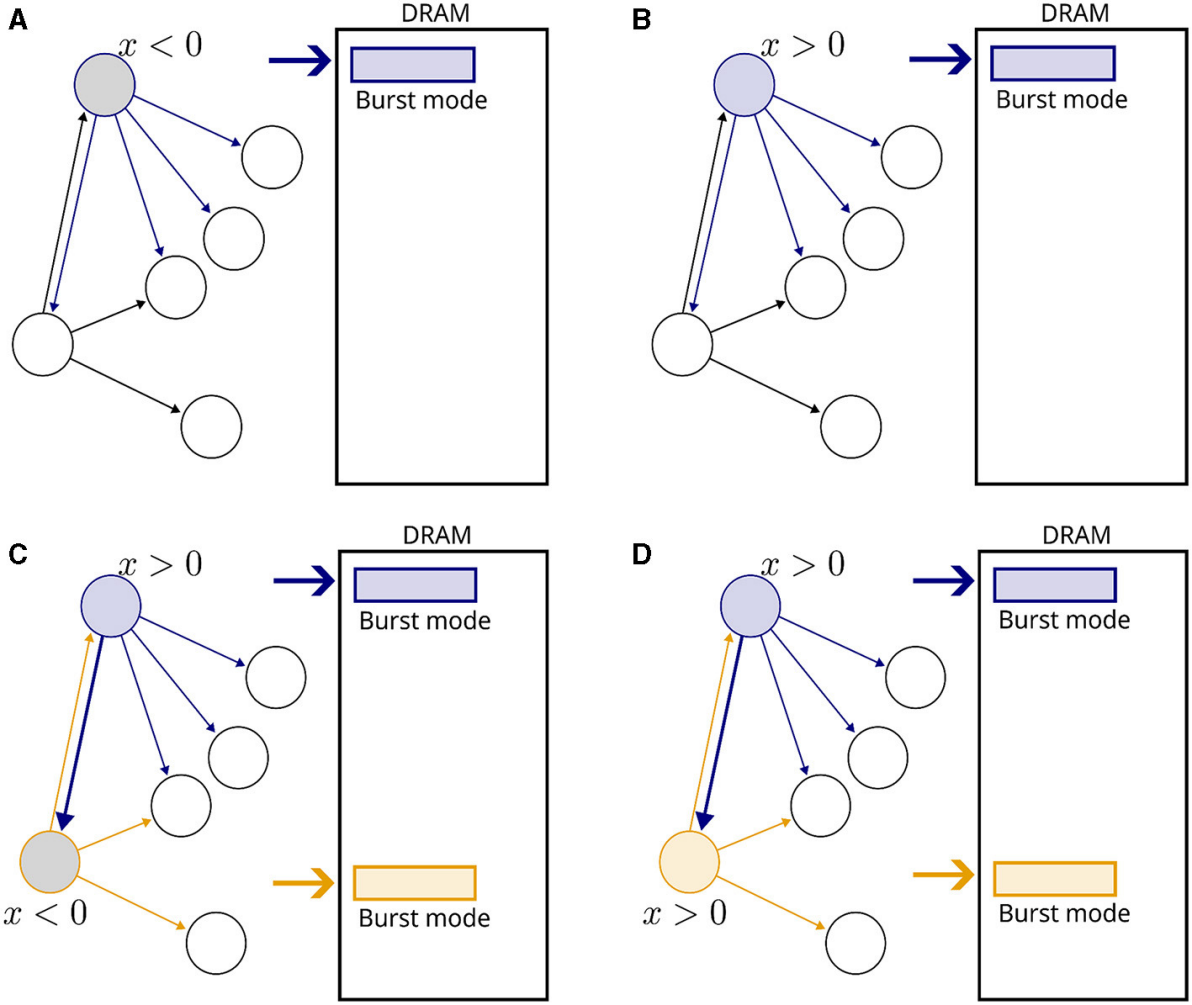
Illustration of the proposed plasticity scheme. Neurons are represented by circles and connections by arrows. Inactive neurons are displayed in black without filling. A rectangle represents a DRAM memory cell. **(A)** A neuron becomes active, indicated by a blue outline. The gray filling means that *x* < 0. All fan-out variables are fetched from the DRAM in burst mode. **(B)** In the next time step, *x* becomes positive and fetching from memory continues. **(C)** Another neuron becomes active, indicated by a orange outline. The connection from blue to orange neurons is potentiated, and new memory fetches are triggered. **(D)** In the subsequent time step, no weight updates take place because both neurons are inactive, but memory fetches continue.

Figure 1 presents a visual illustration. No weight updates are performed when a single neuron is active, but memory fetches of postsynaptic variables are carried out to detect upcoming events (Figures 1A, B). When another neuron becomes active (Figure 1C), a causal update is detected and fulfilled. Note that no reverse memory accesses were necessary. No updates are required in Figure 1D, but memory fetches continue.

**Figure 2:**
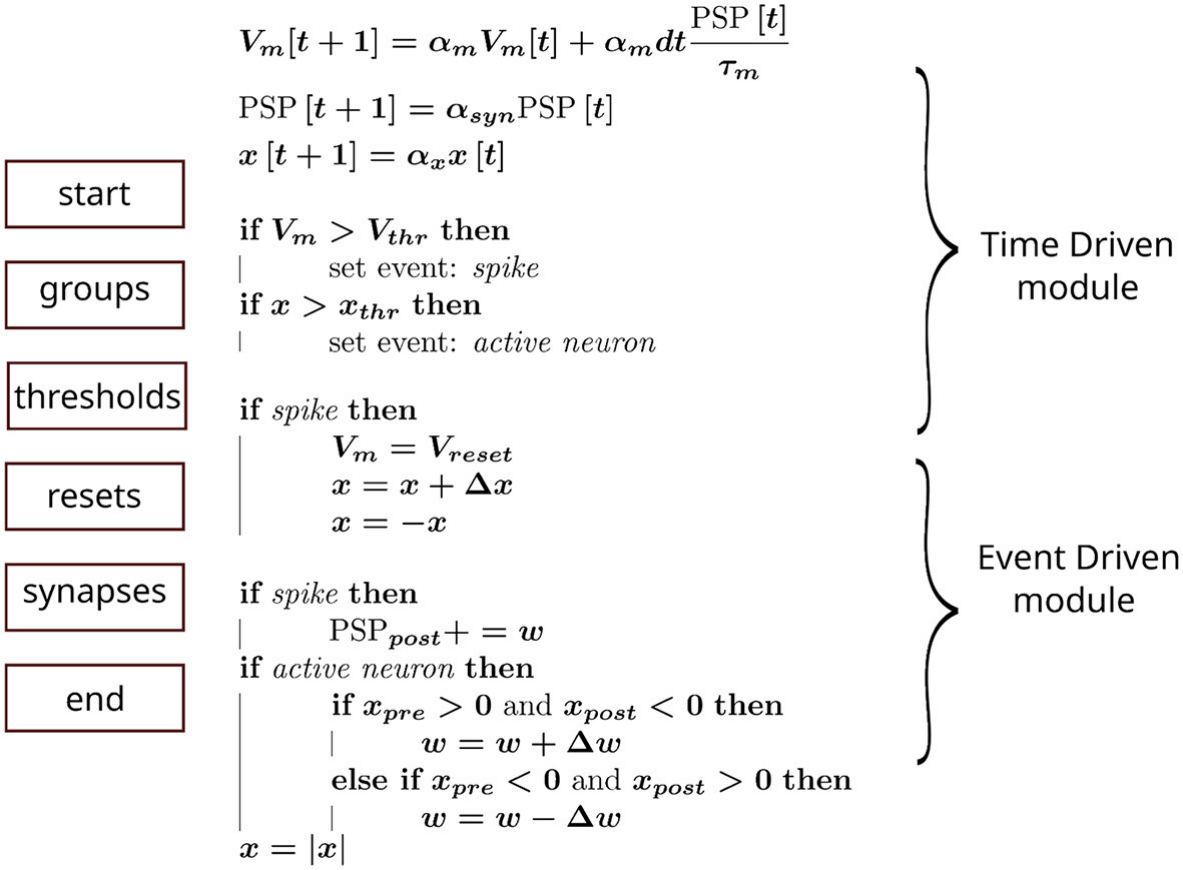
Hardware emulation using Brian2. Each block on the left represents the computation groups available in Brian2. They were scheduled in this sequence (from top to bottom) to approximate hardware behavior. The equations in the middle show the operations performed in each block, whereas the curly braces on the right indicate the corresponding hardware module emulated.

To emulate our simulation pipeline with Brian2, we created a custom event to capture when a neuron was active. This enabled synaptic objects access to state variables and perform the required operations. The general scheme is summarized in Figure 2, where each block on the left column represents the order, from top to bottom, in which Brian2's execution slots were scheduled in a single time step. The calculations assigned to each block are shown in the middle column. Additionally, in the right column, we indicated how this pipeline could be incorporated into an advanced time-multiplexing approach, which splits slots into time-driven and event-driven modules (Wang and van Schaik, 2018).

### 2.3 Simulations performed

To test our approach, we initially simulated simple scenarios in which an input layer of neurons projected onto postsynaptic neurons in a feedforward manner. In these experiments, presynaptic spikes were either generated deterministically or stochastically. The primary objective was to replicate expected outcomes, such as dependence of synaptic modifications on spike timing (Bi and Poo, 1998) and a bimodal distribution of weights (Song et al., 2000). Unless otherwise specified, the parameters related to plasticity were set to the values shown in Table 1.

**Table 1:** Parameters and descriptions of plasticity model.

**Name**	**Value**	**Description**
η	0.1	Plasticity learning rate.
Δ*x*	1	Increment applied to *x* at every spike.
*w*_*max*_	100 mV	Maximum weight value.
τ_*x*_	20 mV	Time constant of *x*.

Conventional models of how a synapse strength is modified through STDP typically incorporate traces for both potentiation and depression. By doing this, it is possible to tune parameters so that synaptic weakening is larger than strengthening, which leads to desirable properties (Song et al., 2000). Our models, however, possess a single trace per neuron, so we relied on other strategies. As heterogeneity is associated with improved stability and robustness (Perez-Nieves et al., 2021), we sampled each τ_*x*_ from a uniform distribution in some of our simulations.

We also investigated and compared the number of memory accesses required for different strategies. While various factors influence memory access in an actual digital system, like latency, locality principle, burst mode, and bandwidth, we simplified the scenario by assuming that accessing a single memory position had a generic cost of 1. This interpretation helps understand the cost of performing STDP in a digital system, especially since reading a single state variable may add many clock cycles of overhead (Pedroni et al., 2019). Clearly, the chosen data structure impacts the access pattern. Therefore, we considered a pointer-based storage structure due to its small memory footprint in sparse, recurrent networks.

The storage cost of the proposed models can be separated into two parts—neurons and synapses. The cost for neurons is calculated as *N*_*var*_*N*_*bits*_*N*_*t*_, where *N*_*var*_ represents the number of state variables (*N*_*var*_ = 3), *N*_*bits*_ is the bit resolution (*N*_*bits*_ = 64), and *N*_*t*_ is the total number of neurons. On the other hand, the cost for synapses can be calculated as *N*_*pre*_ρ*N*_*post*_*N*_*bits*_+*N*_*pre*_ρ*N*_*post*_log_2_*N*_*post*_+*N*_*pre*_log_2_(*N*_*pre*_ρ*N*_*post*_), where ρ is the connection probability. The first term represents the bit resolution of synaptic weights for each connection in the weight table, while the second term depicts the address of the postsynaptic neuron within that table. The last term pertains to the pointer table, which maintains the outgoing connections for each presynaptic neuron.

The computations performed during time-driven and event-driven modules are different, and so is the access cost of each. During the time-driven module, neuronal state variables are loaded, resulting in a cost of 3*N*_*t*_. For the event-driven module, forward access incurs a cost of $N_{pre}^{a}\left(2+\rho N_{post}\right)$, where $N_{pre}^{a}$ is the number of active presynaptic neurons associated with plastic weights (i.e., those whose axons make synaptic connections with other excitatory neurons). Considering that reverse access is achieved by using forward access to traverse weight tables to find the connected neuron pairs, the cost can be expressed as $N_{post}^{a}\left(N_{pre}+N_{pre}\rho N_{post}\right)$. Note that accesses depend on the number of active neurons at every time step, so the size and rate of the neuronal population directly impact these metrics. Moreover, although using fewer state variables per neuron can already decrease storage requirements and memory access overhead, our study concentrated on the memory access complexities related to STDP during the event-driven module.

In our investigation, we compared the access costs associated with our forward-only access strategy and a conventional approach that also includes reverse access triggered by postsynaptic spikes. For the sake of simplification, the computational overhead of fetching the start and end addresses of the weight table was not incorporated into the analysis, as it should be small compared to the weight table itself.

**Figure 3:**
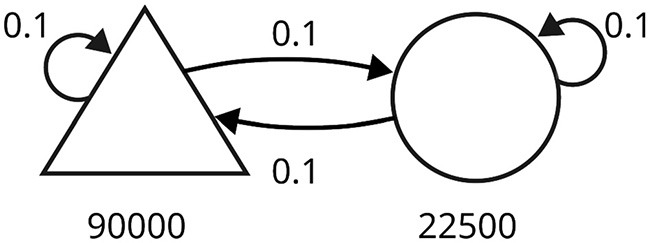
Schematic of the balanced random network model. The triangle represents the excitatory neurons, and the circle represents the inhibitory neurons. The number of neurons in each population is written under the corresponding symbol. Arrows indicate connections between populations, each with a specific probability.

Since we aim to improve the scalability of SNNs endowed with plasticity in digital hardware, we also tested our approach on a large-scale simulation of a balanced network (Morrison et al., 2007), illustrated in Figure 3. The network comprised 90,000 excitatory neurons and 22,500 inhibitory neurons, with a connection probability of 0.1 (represented by the symbol ρ). The number of connections per neuron was about 10^4^, and the total number of synapses in the network was in the order of 10^9^. Neurons were driven by spike trains generated from 9,000 independent Poisson processes at 2.32 Hz.

The plasticity rule in Equation 6 was slightly modified to


(7) $$\begin{array}{l} w_{ji}=\left\{ \begin{array}{ll} w_{ji}-\eta \alpha w_{ji}x_{j} & \text{if }x_{i}<0\wedge x_{j}>0\\ w_{ji}+\eta w_{0}^{1-\mu }w_{ji}^{\mu }x_{i} & \text{if }x_{i}>0\wedge x_{j}<0 \end{array}\right. \end{array}$$

and applied to excitatory-excitatory connections. The additional parameters for the simulation are shown in Table 2. Note that a neuron was not allowed to connect to itself and that there were no instantaneous spike propagations.

**Table 2:** Parameters and description of balanced network with STDP.

**Name**	**Value**	**Description**
**Neurons**		
$V_{m}^{0}$	~N(5.7,7.2) mV	Initial membrane potential.
τ_*r*_	1 ms	Absolute refractory period.
*C*_*m*_	250 pF	Membrane capacitance.
*g*_*l*_	25 nS	Leak conductance.
**Synapses**		
τ_*syn*_	0.66 ms	Time constant of synaptic input.
α	0.1449	Depression strength.
μ	0.4	Power law exponent.
*w*_0_	0.04 mV	Reference weight.
*w*_*exc*_	1 mV	Initial excitatory weights.
*w*_*inh*_	~N(5*w*_*exc*_, *w*_*exc*_/2) mV	Inhibitory weights.
*d*_*e*_	~N(1.5,0.75) ms	Propagation delay of excitatory connections.

The symbol N means a random normal distribution within a given range.

Morrison et al. (2007) implemented their synaptic currents as an α function, which is more complex than the model we adopted. To obtain PSPs similar to their work, we utilized *w*_*exc*_ = 25pA/*g*_*l*_ = 1mV. Moreover, PSPs peaked around 0.14 mV, with a rise time of 1.7 ms and a half-width of 8.5 ms.

## 3 Results

### 3.1 Simple STDP benchmarks

**Figure 4:**
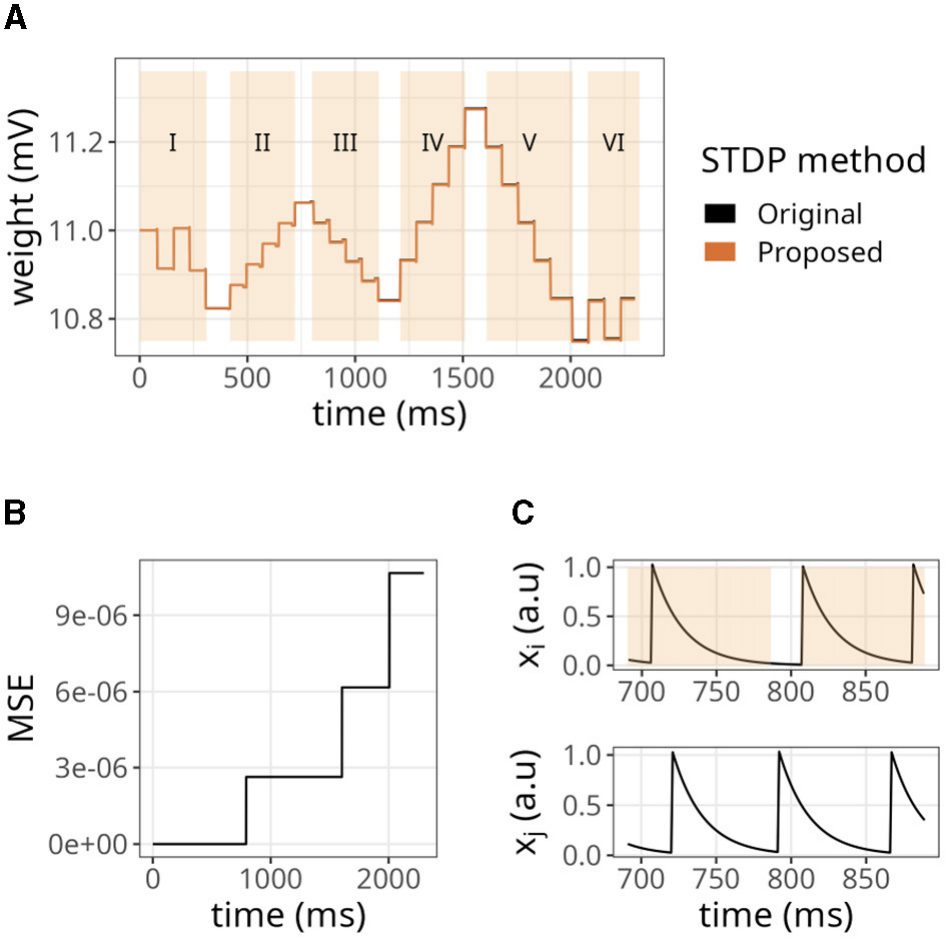
Evolution of state variables in time with the STDP implementation proposed. **(A)** Weight changes of the proposed and original STDP implementations during multiple protocols, namely random (I), weak potentiation (II), weak depression (III), strong potentiation (IV), strong depression (V), and random again (VI). On each protocol, a pair of pre- and postsynaptic neurons were forced to spike in a desired order to elicit the observed updates. **(B)** Mean squared error (MSE) between the original and proposed traces shown in A. The traces in A are very close to one another, so the MSE is used to highlight the small differences between them. **(C)** Traces from pre- (*x*_*i*_) and postsynaptic (*x*_*j*_) neurons, where orange overlay shows regions where the presynaptic neuron was active.

We began by examining a simple scenario where a single synapse underwent multiple potentiations and depressions. Figure 4A illustrates the strength of a synaptic weight over time when multiple STDP protocols (indicated by Roman numerals) were applied. The original and proposed implementations yielded similar values, although minor errors were accumulated over time.

As shown in Figure 4B, the mean squared error (MSE) between them was minimal but increased in the same interval. The difference between our approach and the original formulation lies in the precision of traces. In the original formulation, even if the trace value is minimal, it still causes a slight increase or decrease in the synaptic weight. In contrast, we set a threshold of *x*_*thr*_ = 0.02 in our approach, which means that traces below this value were considered insignificant and the presynaptic neuron was considered inactive. Hence, no weight updates were performed. Figure 4C shows that our approach introduced an error around 791 ms where *x*_*i*_<*x*_*thr*_. As a result, the postsynaptic spike did not trigger any changes in the synaptic weight. Accordingly, a threshold of *x*_*thr*_ = 0, would introduce no errors (not shown).

**Figure 5:**
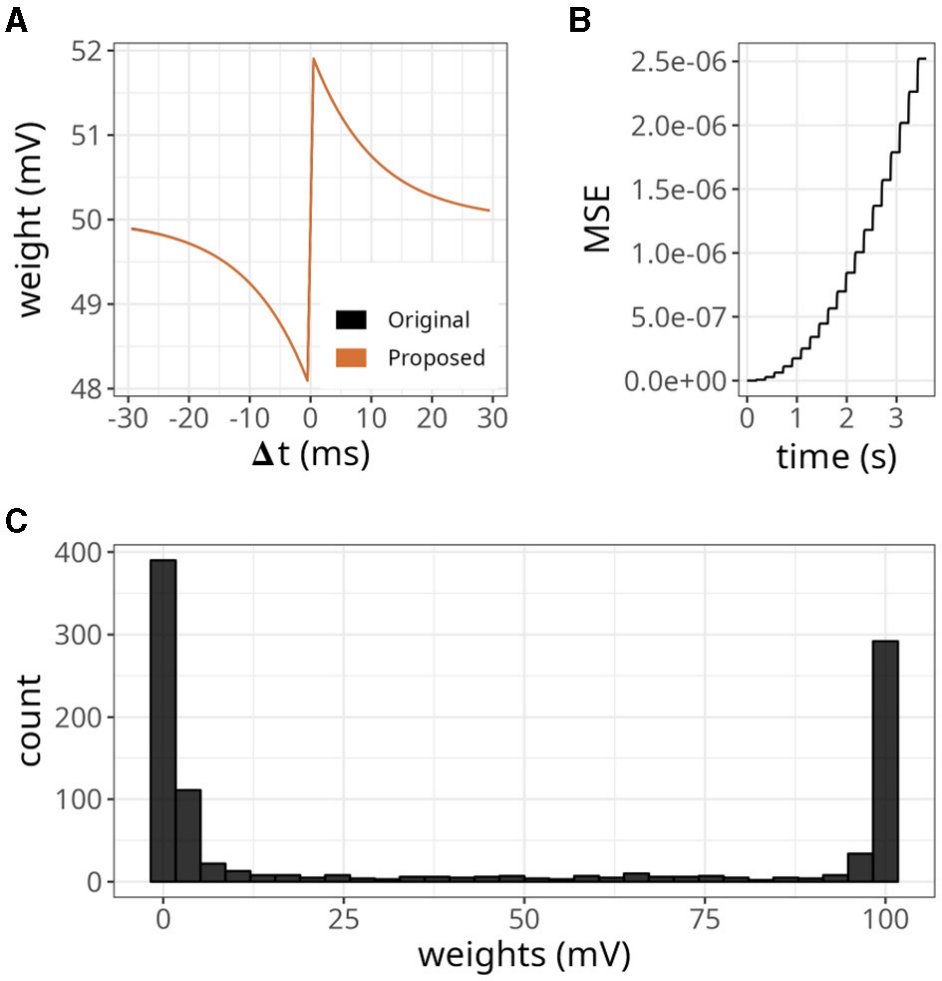
Distribution of weights after undergoing plasticity under the proposed STDP implementation. **(A)** Weight values as a function of the difference between pre- and postsynaptic spikes —Δ*t* = *t*_*post*_ −*t*_*pre*_. Both original and proposed implementations are shown. **(B)** Mean squared error (MSE) between the original and proposed traces shown in **(A)**. **(C)** Bimodal distribution of weights under the proposed STDP implementation.

To further evaluate the STDP proposed, we replicated some established properties of this plasticity rule. Figure 5A shows the dependence of synaptic modification on spike timing. For the experiment, we connected each pair of neurons with an initial weight value of 50 mV, and a fixed spike timing between them was repeated 100 times. The results were similar to the original formulation. Nevertheless, as shown above, minor errors accumulated (see Figure 5B).

In our STDP implementation, the distribution of plastic synaptic weights in a network with 1000 presynaptic neurons firing at 15 Hz and one postsynaptic neuron converged to a bimodal distribution. This is illustrated in Figure 5C. Most of the weights were either close to zero mV or close to the maximum value of *w*_*max*_ = 100 mV. It is worth noting that the selected values of τ_*x*_ played a significant role in shaping the distribution profile. The range of τ_*x*_ values used in the above results varied between 5 and 15 ms and were randomly sampled from a uniform distribution. The initial weights were drawn from a gamma distribution with *k* = 1 and θ = 17.5. However, the initial weight values did not significantly impact the outcome as long as the postsynaptic neuron was firing.

### 3.2 Efficiency measurements

In the previous simulations, we observed that reducing the value of *x*_*thr*_ led to fewer deviations from the original STDP formulation. However, it also increased the number of memory accesses performed at every time step. To further understand the impacts of different threshold values, we calculated the number of memory accesses required for the bimodal distribution benchmark, which highlights some desirable properties of STDP. Additionally, we set the value of *w*_*max*_ to 0.4 to limit the final firing rate of the postsynaptic neuron. Since τ_*x*_ can affect the distribution of weights, we adjusted its interval to be between 16 and 26 to ensure a bimodal distribution.

**Figure 6:**
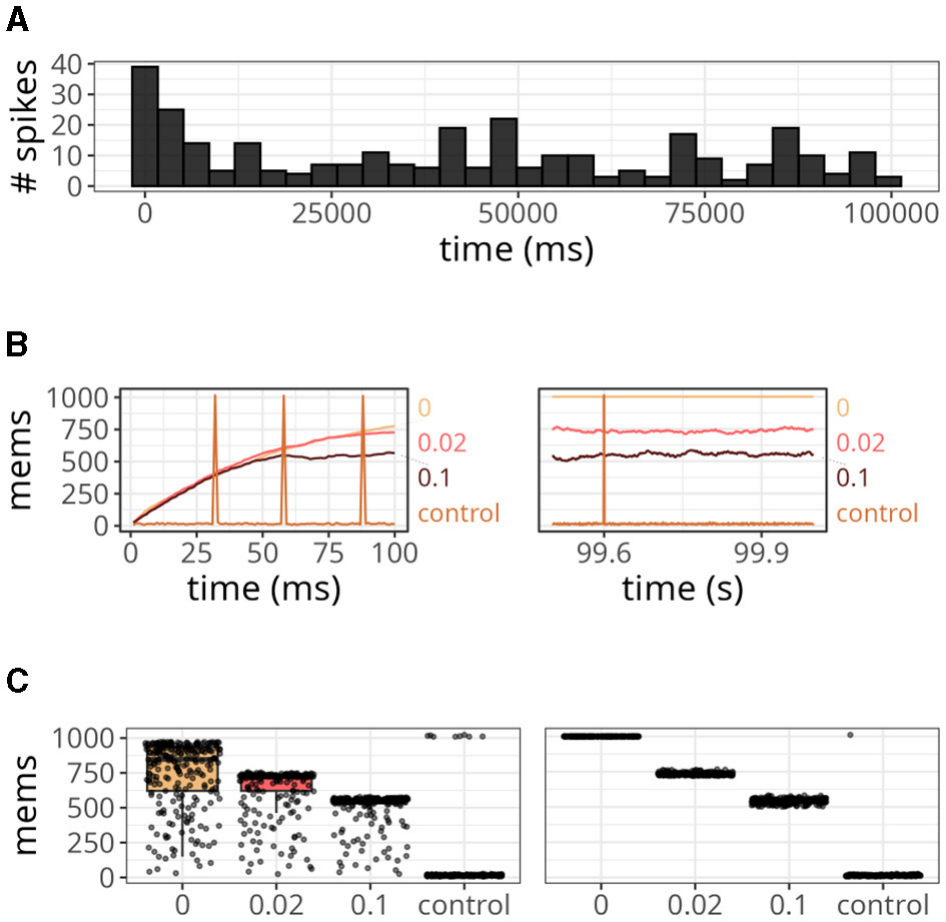
Analysis of the number of memory access (mems) over time. **(A)** Histogram of spike counts of the postsynaptic neuron over time. **(B)** Number of memory accesses at each time step for different implementation strategies. The time interval considered ranged from 0 to 100 ms on the left panel, and from 99.5 to 100 s on the right panel. **(C)** Boxplots of memory fetches for each time step. Individual data points are also shown on top of the boxes. The time interval considered ranged from 0 to 250 ms (left panel), and from 99.5 to 99.75 s (right panel).

The results are displayed in Figure 6. Our approach, with various *x*_*thr*_, was compared to a conventional formulation (i.e., forward and reverse access) labeled as “control.” At the start of the simulation, the number of spikes emitted by the postsynaptic neuron reached values close to 40 but gradually decreased over time (Figure 6A). This is a relevant observation because when the postsynaptic neuron is not spiking, the number of memory accesses is determined by all active presynaptic neurons driving forward accesses.

Since we connected all 1,000 presynaptic neurons firing at 15 Hz to a single postsynaptic neuron, the control case shown in Figure 6B displayed around 15 memory accesses at most time steps. However, postsynaptic spikes caused peaks that reached values slightly above 1,000 due to reverse access. In contrast, our approach was not affected by postsynaptic spikes, but it yielded numbers of memory accesses higher than the control case. When we set *x*_*thr*_ = 0, neurons could not become inactive (i.e., *x* = *x*_*thr*_) due to the high precision of double-precision floating-point numbers. Therefore, the number of memory accesses in this case increased persistently until the maximum, which was 1,000. As we increased *x*_*thr*_ (e.g., to 0.02 or 0.1), more neurons became inactive, causing the number to converge to a smaller value at the end of the simulation (see right panel of Figure 6B).

In Figure 6C, we show the boxplots of memory fetches for each time step over 250 ms at the start and end of the simulation. From the data, it appears that the effects of reverse access are not significant in the control case. However, it is worth noting that the previous simulations only involved low spiking rates and a single postsynaptic neuron.

**Figure 7:**
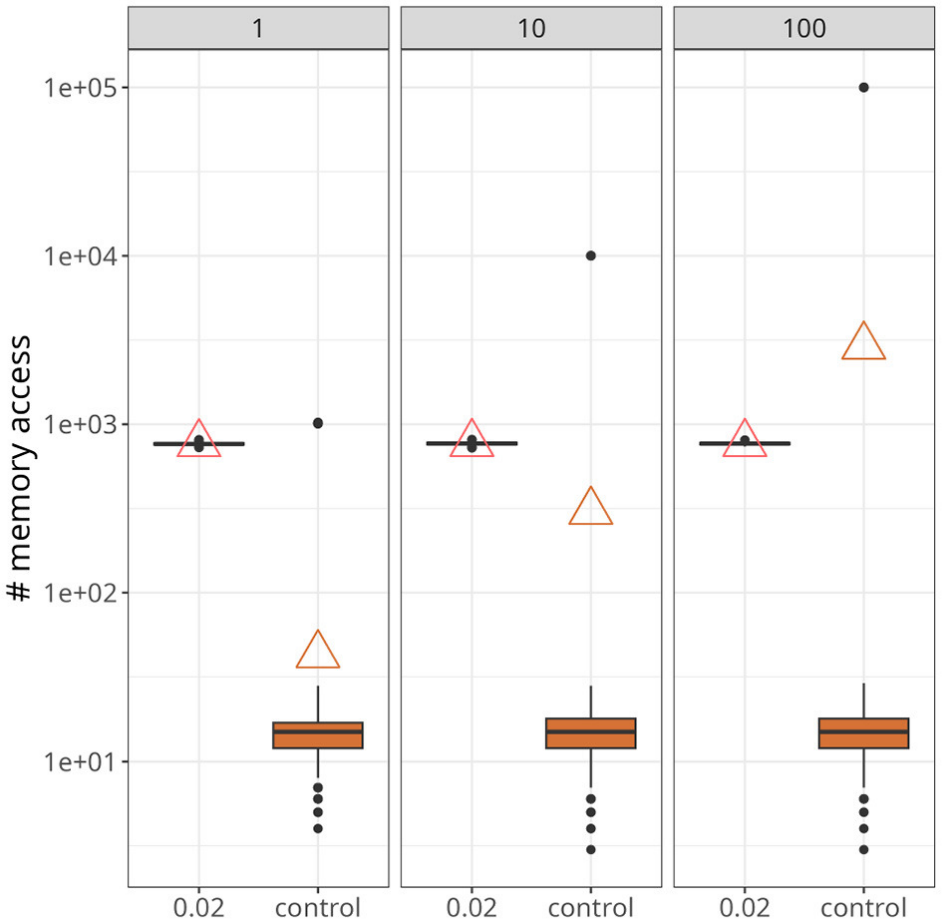
Statistics of memory accesses. Each boxplot was calculated based on ten simulation runs. Triangles denote the average memory access in each case. The number at the top of each panel represents the number of postsynaptic neurons. In all panes, our approach, with *x*_*thr*_ = 0.02, was compared with the conventional (i.e., control) formulation.

Figure 7 demonstrates a scenario with a higher number of postsynaptic neurons and slightly higher firing rates, around 25 Hz. The data points were taken from a time interval of 250 ms after both traces converged to a stable value. Upon closer inspection, it becomes apparent that our approach was not affected by the increase in postsynaptic neurons (indicated at the top of each panel). On the other hand, for the control case, not only did the maximum value increase significantly as the number of postsynaptic neurons increased, but also the average. In fact, with 100 postsynaptic neurons, the average value of the control case was higher than the proposed approach.

### 3.3 Large-scale networks

**Figure 8:**
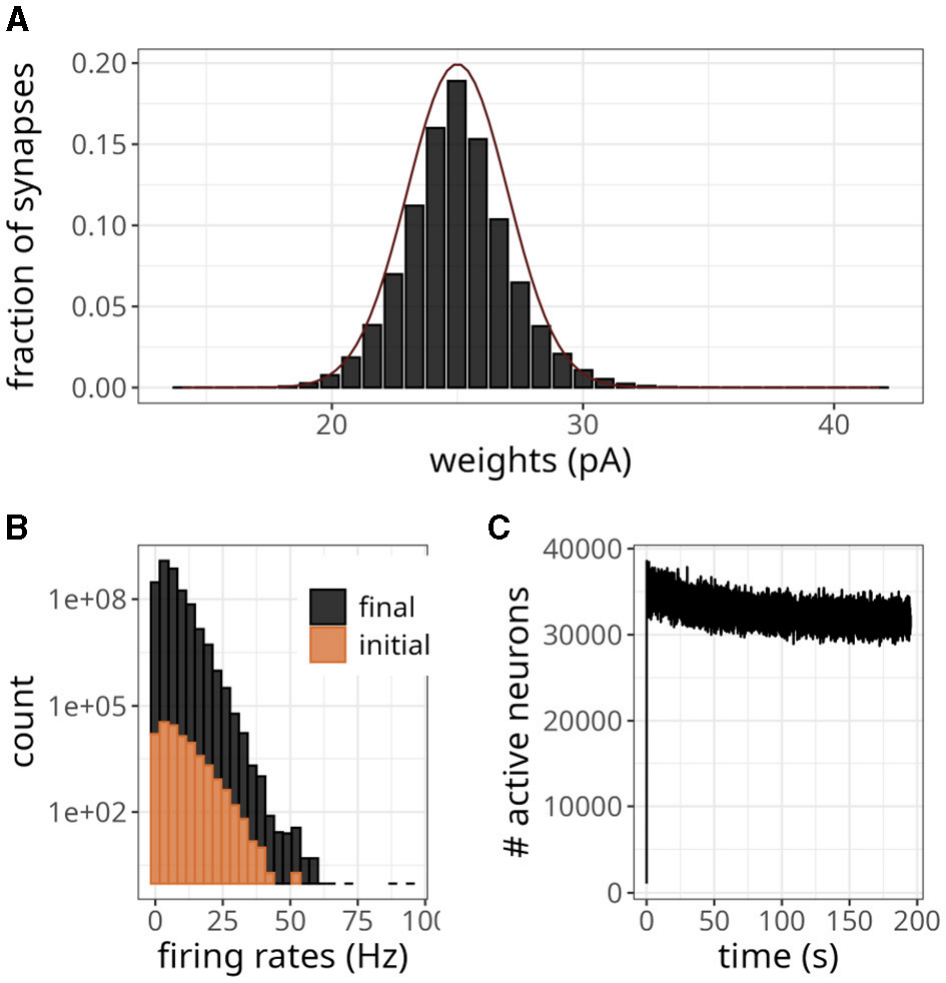
Large-scale balanced network with plasticity. **(A)** Histogram of synaptic weights. The dark line represents a Gaussian distribution with a mean of 25 and a standard deviation of 2. **(B)** Distribution of firing rates across neurons in the initial and final 2 s. **(C)** Number of active neurons over time for plasticity updates.

As excitatory weights in a network increase due to STDP updates, the stability of the system can be compromised. This can be particularly problematic in large networks, where each neuron makes thousands of connections. In Figure 8A, we have shown the final weight distribution (i.e., after 200 s) of a large-scale network endowed with the plasticity rule of Equation 7. To facilitate comparisons with the original model (Morrison et al., 2007), we multiplied our weights by *g*_*l*_ to get values in pA. Although the initial value of those weights was 25 pA, they evolved to form an unimodal distribution. The final shape was similar to a normal distribution with a mean of 25 and a standard deviation of 2. Note that the maximum synaptic strength in these experiments was set to *w*_*max*_ = 1, 000 mV, which indicates that the weights settled to a stable value without saturation.

The statistics of the network were akin to an asynchronous and irregular regime, with a mean firing rate of 5.70 Hz and CV of 0.90. The spike variability was high, as indicated by a Fano factor of 5.11. The histogram in Figure 8B shows that only a few neurons exhibited high firing rates, even though the weights were small. The difference in count values suggests that regular activity of the network at the beginning of the simulation produced an intense blanket of inhibition, effectively silencing some neurons. As irregularity increased, other neurons became more susceptible to excitatory drive, yet the average number of active neurons revealed a decreasing trend (Figure 8C).

According to the magnitude of the network analyzed, the storage cost associated with neurons and synapses is around 2.7 MB and 12.78 GB, respectively. Figure 8C illustrates the pattern of active neurons over time, which shows an initial peak followed by a steady decrease. This number fluctuated around a mean of ~32,000 per time step, probably as a result of the high activity levels of some neurons (see Figure 8B). Nevertheless, this number is lower than the worst-case scenario of 90,000.

**Figure 9:**
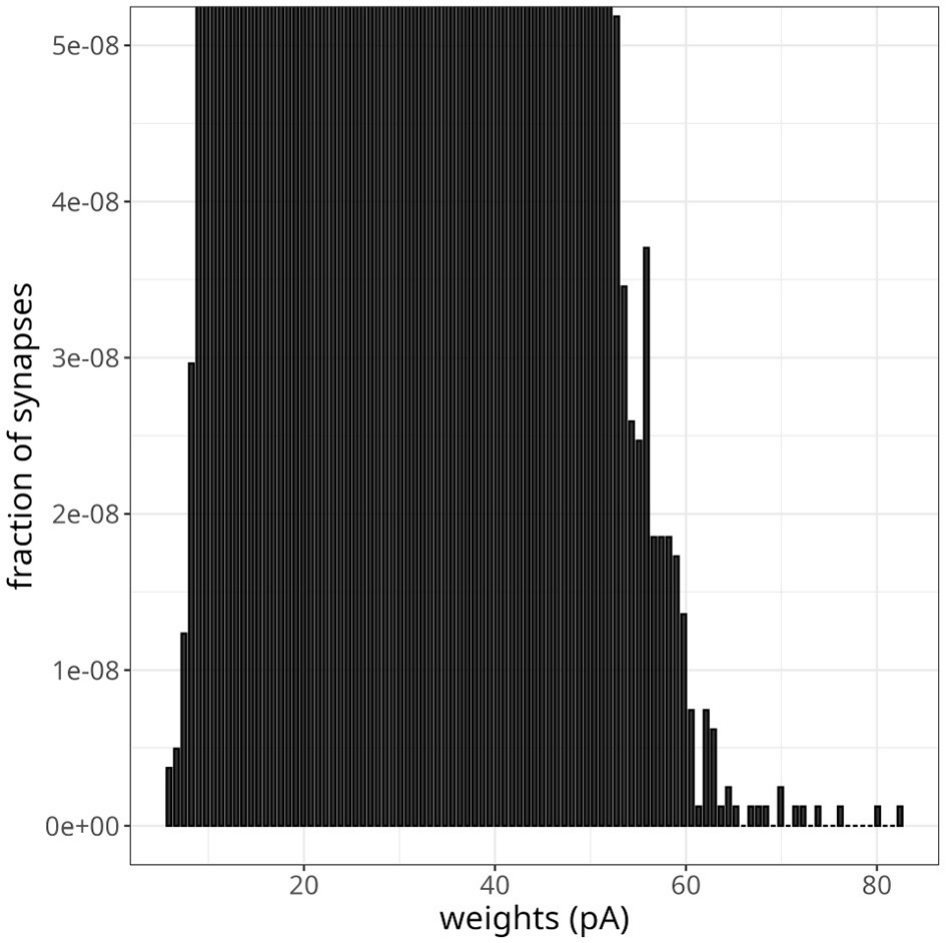
Instability in balanced network with plasticity. Histogram of synaptic weights after a simulation time of 13 s. The plot was zoomed in to visualize higher-weight but less frequent synaptic connections.

As pointed out by the authors who first proposed Equation 7, if the parameter α was slightly smaller than $\alpha_{p}={\frac{w^{*}}{w_{0}}}^{\mu -1}$, where w^*^ is the fixed point of the synaptic weight distribution, depression would not be able to counteract strong potentiations induced by fast oscillations effectively. To test this scenario in our model, we decreased α by 2%, going from α = α_*p*_ = 0.1449 to α = 0.1420. Figure 9 shows the resulting weight distribution after 13 s of simulation. Most weights were still concentrated around the mean of 25 pA, but a small proportion of synapses were further strengthened, with weights extending up to 82 pA. Despite the emergence of some denser regions suggesting clustering, these highly potentiated synapses were predominantly dispersed and lacked a discernible pattern. Simulating for 20 s resulted in even stronger weights, with a small fraction sparsely distributed between 60 pA and the maximum weight of 25 nA.

**Figure 10:**
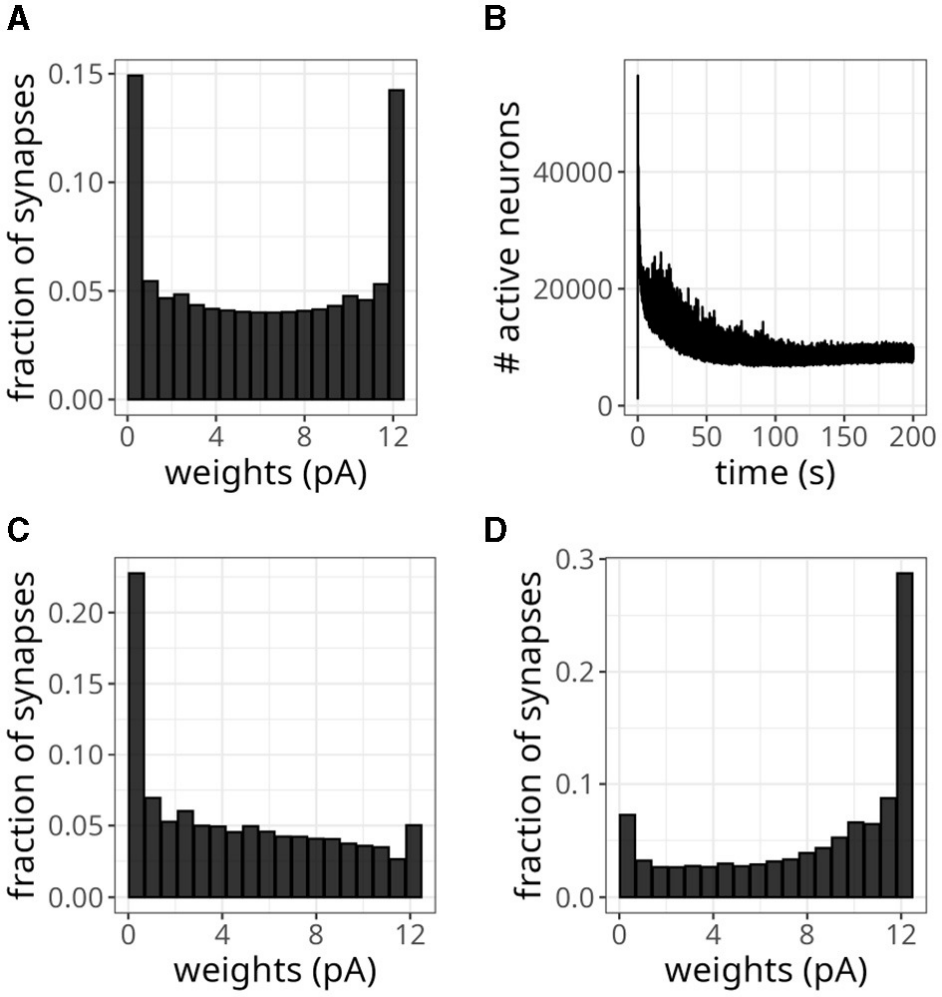
Balanced network with alternative plasticity rule. **(A)** Bimodal distribution of weights. **(B)** Number of active neurons over time with plasticity rule in Equation 6. **(C)** Distribution of weights projecting to neuron 0. **(D)** Distribution of weights projecting to neuron 10,000.

We wanted to verify whether our model could replicate a bimodal distribution, so we replaced the previous plasticity rule with Equation 6. Figure 10A shows the resulting bimodal profile. Although intermediary values were not negligible, we observed prominent peaks close to the minimum and maximum values. To generate this distribution, we set the maximum weight to *w*_*max*_ = 0.5 mV and sampled the initial *w*_*exc*_ from a random uniform distribution. To sample the inhibitory weights as in Table 2, we selected the reference excitatory weight as *w*_*exc*_ = 0.25 mV. We could have adopted a higher cap without compromising this weight distribution, but we wanted to avoid high firing rates.

The average activity rate recorded was 2.31 Hz, with the maximum rate not exceeding 29 Hz. Moreover, neurons exhibited irregular discharges, as indicated by a CV of 1.30. The pattern of active neurons over time, shown in Figure 10B, displayed a decreasing trend over time. Eventually, it plateaued at a lower level than the unimodal distribution experiment due to the smaller rate.

Instead of using a fixed value of 20 ms, randomly sampling τ_*x*_ from a uniform distribution between 10 and 25 ms introduced more heterogeneity in the distribution of incoming synaptic weights. Namely, besides bimodal distributions, we observed profiles skewed to the left for some neurons and to the right for others. Figures 10C, D display this difference for synaptic connections projecting into neurons 0 and 10,000, respectively. Despite this variability, the distribution of all the plastic weights in the network remained bimodal, as in Figure 10A.

## 4 Discussion

When emulating a multitude of neurons and synapses, scalability is a common theme (Thakur et al., 2018). As the scales reach massive levels, several optimizations are necessary to make the simulation platform as efficient as possible. In this paper, we proposed a scheme to facilitate large-scale implementations of SNNs endowed with STDP on digital neuromorphic platforms. We replicated desirable properties resulting from the plasticity rules studied while also considering deviations from expected outcomes, which can be controlled through a parameter. For tasks that depend on STDP and require high accuracy, we could set *x*_*thr*_ = 0 to obtain a conventional STDP formulation.

The main issues tackled were communication bandwidth and scalability, which are significant underlying challenges, particularly when real-time is concerned (Wang et al., 2018). Our approach emphasizes the synergy between computational primitives to foster better usage of resources in a digital design (Cassidy et al., 2013; Frenkel et al., 2023). Moreover, our general approach is compatible with an advanced time-multiplexing strategy (Figure 2), which eliminates the need for buffering a high number of events generated in a large SNN (Wang and van Schaik, 2018).

A simple implementation of STDP involves modifying pre- and postsynaptic plasticity traces or times per synapse every time a spike occurs. While this ensures proper spike propagation and weight update, it increases memory footprint, especially as the number of synapses increases. Additionally, this overlooks the redundancy of the data stored: if a neuron spikes at time *t*, synapses associated with that neuron are bound to replicate the same temporal information (time or trace) across synapses (Cassidy et al., 2013; Davies et al., 2018). There are instances when this is done intentionally, such as when each synapse has a different decay rate (see Song et al., 2000), but this is not always made explicitly clear in digital designs.

In our case, we improved scalability by implementing a single plasticity trace per neuron. This approach is similar to well-known methods that reduce computations relating to the multitude of synaptic conductances in large networks (Lytton, 1996; Brette et al., 2007). Intriguingly, STDP has been described as a consequence of the dynamics of one specific biochemical messenger: intracellular calcium concentration (Shouval et al., 2010; Graupner and Brunel, 2012). Although we did not explore this alternative plasticity mechanism, it would be an interesting expansion of our models. Different phenomenological models can be developed by tuning time windows or adding extra state variables (Pfister and Gerstner, 2006; Clopath et al., 2010). Therefore, we anticipate that implementing other plasticity rules should be straightforward.

In this work, we extended the functionalities of this single trace to get more benefits from this implementation. Since a negative value of our trace represents the occurrence of a spike, we do not rely on FIFOs or bitmaps to buffer pre- and postsynaptic spike times. As a result, events can be processed at the appropriate time slot while reducing the area required. An efficient digital design would involve storing a matrix containing all the active neurons. On-chip memory can store this information, as only a single bit is needed per neuron. Once the outgoing connections for each neuron are fetched, their weights can be updated accordingly with only forward memory accesses.

On-chip memory cells are limited in size, so it is expected that some data may need to be stored in an off-chip DRAM. This is the case for the large-scale network presented here, where we estimated that the memory necessary to store the synaptic weight table was 12.78 GB. In this circumstance, efficient memory controllers are required to deal with communication bandwidth and bolster high throughput (Cassidy et al., 2013; Wang et al., 2018), particularly for the memory-intensive task of simulating many neural elements in real time. Our weight update scheme can facilitate this process because no reverse accesses are required, and the outgoing synapse weights of a neuron can be stored close together. This results in increased throughput, as burst reads in DRAMs can efficiently retrieve sequential data blocks from memory in fewer access operations. By doing this, we avoid increasing silicon area to accommodate more (or more complex) memory cells (Seo et al., 2011; Knight and Nowotny, 2018; Bautembach et al., 2021).

As shown in Figure 7, the average number of memory fetches in the control case (i.e., with reverse access to the weight table) increased with the number of postsynaptic neurons. With only 100 postsynaptic neurons, it became higher than our proposed scheme. This result may seem counter-intuitive because we fetch state variables as long as the plasticity trace is higher than a threshold, contrasting with memory fetches occurring only when a spike is emitted. The problem, however, is that reverse access elicited by postsynaptic spikes is costly, and there are multiple reasons why it would be worse if more realistic scenarios were incorporated. First, large and recurrently connected networks imply a much greater number of postsynaptic spikes. Second, as demonstrated in Figures 8C, 10B, there is a period of higher activity that persists before plasticity can (potentially) establish a regime with sparser activations. Finally, experimental evidence suggests a skewed distribution of firing rates (Buzsáki and Mizuseki, 2014), which culminates in a small number of highly active neurons, as seen in Figure 8B. Therefore, we argue that our strategy is suited for simulations involving these factors.

It is worth noting that other previously reported networks were larger and more complex than the one we tackled (Schmidt et al., 2018; Wang et al., 2018; Yang et al., 2018). Still, our experiment showed how the communication bottleneck constrains the system, considering it has about one billion connections. We reported a few megabytes associated with time-driven module operations. Memory access of synaptic connections during event-driven module is the main limiting factor in performance, as the computations performed are relatively simple. Taking the unimodal distribution protocol as a reference, the average number of active neurons at every time step was around 4 × 10^4^. This value is higher than that shown in Figure 8C because all neurons (i.e., inhibitory and excitatory) were considered. With an average of 10,000 outgoing connections per neuron, we obtain an estimated 400 G memory accesses per second. Although not discussed here, the bit resolution adopted is clearly a limiting factor. Using double-precision enabled us to use reasonable values for all network variables (e.g., weights and time constants). However, this could easily compromise the real-time capabilities of the digital design. In fact, many neuromorphic systems are limited to much smaller resolutions (Diehl and Cook, 2014; Davies et al., 2018; Frenkel et al., 2018; Wang and van Schaik, 2018). Doing so could still result in high data rates (e.g., 400 GB/s if weights have 8 bits), but recent memory technologies such as High Bandwidth Memory provide a promising prospect; their maximum theoretical bandwidth is ~460 GB/s and has been reported to achieve 406.6 GB/s with burst length of 15 (Pedroni et al., 2020).

Our solutions share similarities with the work of Pedroni et al. (2019). Namely, we also organized weights in a fan-out manner and introduced an alternative event—distinct from a spike—to signal when postsynaptic state variables should be accessed and potentially updated. However, in contrast with their approach, we did not utilize the onset and expiration of timers to elicit updates; Instead, a neuron produced updates as long as it was active, that is, when its plasticity trace *x* fulfilled *x*>*x*_*thr*_. This results in more frequent memory fetches, but there are certain considerations to be made. For instance, when simulating SNNs, it is common to emulate neurons with a 2 ms refractory period. Additionally, trace values are generally only truncated after a few time constants have elapsed to preserve temporal information (e.g., for τ = 20 ms, *x* decays to about 5% of its initial value only after 60 ms). Performing STDP in a timer-based framework would require ⌊60/2⌋ = 30 timers, each with 2 bits. In terms of storage cost, that would nearly equate to the size of a double-precision floating-point number. On the other hand, our method requires no extra traces in such scenarios, and the bit-precision of traces could be reduced while avoiding significant impacts on the temporal profile of the decay. Regarding memory access, performing updates at the onset and expiration of timers would yield the same number of memory accesses as we observed.

Our study is unique in that we focused not only on plasticity but also on large network models and their statistics. In forming a bimodal distribution in conventional models, the profile emerges because depression is initially favored, enforced by the height or duration of the plasticity window. This usually means that at least two variables are required: one for potentiation and the other for depression. However, we achieved the same outcome with a single trace by randomly sampling each time constant. We did not attempt to identify the spontaneous formation of neuronal groups (Izhikevich et al., 2004), but we expect them to emerge given the delay and weight distribution employed. In our examination of an unimodal distribution, we obtained the expected profile despite having synaptic currents that could make neurons more sensitive to synchronous activity. Because of that, the final mean weight and its standard deviation were different but with similar proportions. However, the statistics are expected to differ, as indicated by the lower rate and fano factor. Finally, we also showed that the network was operating close to a bifurcation point and that a slight decrease in the depression factor for a given weight equilibrium led to unstable synaptic growth.

Previous studies have demonstrated large-scale networks with more detailed conductance models (Yang et al., 2018), contributing to our understanding of how different levels of complexity interact. In our approach, however, we chose to favor scale instead of this level of synaptic realism. Indeed, Yang et al. (2018) managed to emulate around 60 million synapses, whereas around 1 trillion synapses were reported by Wang et al. (2018). Of course, simplifications such as the ones we described here are not always desired, but they provide a framework for when massive connectivity levels are the primary focus. Accordingly, we did not consider using crossbars for synaptic connectivity data, as it is not the most effective way of storing recurrent and sparse weight tables (Pedroni et al., 2019). We also did not double weight tables to tackle backward access, which would hurt scalability.

## 5 Conclusion

In conclusion, we explored a framework to enhance the scalability of large-scale SNNs, focusing primarily on neural models and memory access optimization. Further research will involve integrating this methodology with low-precision data types and examining their combined impact on both the efficiency and the accuracy of simulations. This integrated approach offers a comprehensive perspective on optimizing SNNs regarding resource management and computational effectiveness.

## Data Availability

The original contributions presented in the study are included in the article/supplementary material, further inquiries can be directed to the corresponding author.
